# A Word to Hospital Critics

**Published:** 1898-04-02

**Authors:** 


Vol. XXIV.—No. 601.] Edited by Sir Henry Burdett, K.C.B., and Solomon C. Smith, M.D., M.R.C.P. [April 2, 1898.

### A Word to Hospital Critics.

The question of hospital reform has been set back rather than advanced by the circumstance that those who have recently made themselves most active in advocating reform do not, in fact, possess a practical knowledge of the subject with which they have identified themselves. If they would recognise this, and by patient labour acquire an intimate knowledge of the condition and circumstances of each of the hospitals, especially of the metropolitan hospitals, we should welcome their co-operation, and look forward with hope to the results which might then be secured. As things are at present, the new criticism lacks force, and the new critics deservedly fail to attract to themselves the support without which their efforts must be void of result. The strongest confirmation of the necessity for pointing out these truths is the fact that a meeting recently held, which was reported at length, and treated as a representative and important gathering by some of our contemporaries, consisted, as we stated in The Hospital, of a meeting of four and ultimately of nine persons, including the chairman and honorary secretary. We fully recognise the zeal of the few men who, with the best motives, have set themselves to the task of dealing with hospital reform; but such efforts must prove mischievous rather than helpful unless the organisation is recast so as to secure that no statement shall be put forward unless it is supported by practical knowledge, the result of laborious inquiry and conscientious study of the whole of the questions to be dealt with. What can be said of an inquiry into the condition of the out-patients attending a hospital where the inspector's visit occupies about five minutes and the out-patients number upwards of 100 per diem!

We have from time to time, and for many years, called attention to the evils connected with the multiplication of special hospitals; but the time has arrived when, in justice to these institutions, it should be pointed out that the best-managed special hospitals have distinct merits of their own which are worthy of the attention of the authorities of the great hospitals. It is, for instance, important that we seem to have arrived at a period when the number of patients attending the special hospitals has attained something like a maximum, and the probable causes of this result are worthy of study. It is not sufficiently recognised that during the last quarter of a century very few special hospitals have been founded in the metropolis, that these only amount to twelve in number, and that of this twelve four are for children, three are local institutions for lying-in women, and the remaining five, with one doubtful exception, are unimportant and unnecessary. It is manifestly unfair to treat the thirty special hospitals, which have been established upwards of twenty-five years, and many of which have done work of the greatest importance, not only to the public, but to the profession, as if they belonged to the group of five useless or unnecessary institutions which have been established during more recent years. Some of the older special hospitals are as well managed and as conscientiously administered as any voluntary charities in the world.

What, then, is the cause which has placed certain of the best managed special hospitals in the position of something like finality, so far as the number of out-patients attending each year is concerned? It is due, we believe, to the system of enquiry which prevails at these institutions in regard to the out-patients. That system is based upon the American principle that no person is entitled to free medical relief if they can afford to give something towards the cost of their treatment at the hospitals. No doubt it is urged by a few unpractical persons that it would be an evil thing for the public to get it into their heads that the system of subscribing to hospitals might be modified, so far as the humbler citizens are concerned, so as to provide that their contributions should be regarded rather as a matter of insurance than of charity. But on the other hand, if the people could be taught to be provident, if they could be made to understand that it was a sensible thing for them to give up some small luxury in order to be able to give in small annual subscriptions a portion of the cost of their treatment at the hospitals, the whole character of the people concerned must materially benefit. The Prince of Wales's Hospital Fund, by the institution of the subscription books and stamp albums, has placed it in the power of the hospital authorities to reconstruct their system of out-patient relief by placing it within the means of patients to become annual subscribers of small sums to the institutions to which they owe so much. The movement is at present in its infancy, but we believe that it is one which deserves to succeed, because its successful institution must tend to diminish hospital abuse, to legitimately increase the resources of the hospitals, and to add to the self-respect of the citizens who are compelled in existing circumstances to depend upon the hospitals for medical aid, for which they are unable to pay an adequate sum.

